# Antimicrobial Resistance in Veterinary Bacterial Pathogens: Resistance Patterns, Zoonotic Risks and One Health Implications

**DOI:** 10.3390/pathogens15050525

**Published:** 2026-05-13

**Authors:** Ionela Popa, Ionica Iancu, Sebastian Alexandru Popa, Alexandru Gligor, Kalman Imre, Emil Tîrziu, Timeea Bochiș, Călin Pop, Janos Degi, Andrei Alexandru Ivan, Michael Dahma, Ana-Maria Plotuna, Marius Pentea, Viorel Herman, Ileana Nichita

**Affiliations:** 1 Department of Semiology, Faculty of Veterinary Medicine, University of Life Sciences “King Mihai I” from Timisoara, 300645 Timisoara, Romania; ionela.popa@usvt.ro (I.P.); timea.bochis@usvt.ro (T.B.); calinpop@usvt.ro (C.P.); 2 Doctoral School “Veterinary Medicine”, University of Life Sciences “King Mihai I” from Timişoara, 300645 Timisoara, Romania; andreialexandru.ivan.fmv@usvt.ro (A.A.I.); michael.dahma@usvt.ro (M.D.); anamaria.plotuna@usvt.ro (A.-M.P.); 3 Department of Infectious Diseases and Preventive Medicine, Faculty of Veterinary Medicine, University of Life Sciences “King Mihai I” from Timisoara, 300645 Timisoara, Romania; ionica.iancu@usvt.ro (I.I.); janosdegi@usvt.ro (J.D.); viorelherman@usvt.ro (V.H.); 4 Department of Animal Production and Veterinary Public Health, University of Life Sciences “King Mihai I” from Timisoara, 300645 Timisoara, Romania; alexandru.gligor@usvt.ro (A.G.); kalmanimre@usvt.ro (K.I.); emiltirziu@usvt.ro (E.T.); ileananichita@usvt.ro (I.N.); 5 Department of Anatomy, University of Life Sciences “King Mihai I” from Timisoara, 300645 Timisoara, Romania; mariuspentea@usvt.ro; 6 Academy of Romanian Scientists, Str. Ilfov Nr. 3, Sector 5, 050044 Bucharest, Romania

**Keywords:** antimicrobial resistance, veterinary medicine, One Health, multidrug resistance, zoonotic transmission, livestock, companion animals

## Abstract

Antimicrobial resistance (AMR) has emerged as one of the most significant global health challenges affecting both human and veterinary medicine. The growing prevalence of resistant bacterial strains in livestock and companion animals not only compromises treatment efficacy but also poses serious public health risks through potential zoonotic transmission. Recent molecular and genomic studies have shown the widespread dissemination of resistance genes across different ecological compartments, emphasizing the need for integrated monitoring systems. Antimicrobial stewardship programs and evidence-based interventions are therefore essential in veterinary medicine to mitigate these trends. This is particularly important because the emergence of multidrug-resistant (MDR) pathogens is increasingly associated with mobile genetic elements, such as plasmids, transposons, and integrons, which facilitate horizontal gene transfer within and across bacterial species.

## 1. Introduction

Throughout history, human populations have been engaged in a persistent struggle with microorganisms, particularly bacterial pathogens, which have contributed substantially to global patterns of morbidity and mortality across diverse demographic groups [1]. Antimicrobial agents constitute a fundamental component of veterinary practice, being widely employed for the treatment and management of bacterial infections in animal populations [2]. Antimicrobial resistance (AMR) is the ability of microorganisms, including bacteria, to survive and multiply in the presence of antimicrobial agents that would normally inhibit their growth or kill them [1].

AMR represents one of the most pressing challenges facing modern medicine. The rapid emergence and dissemination of resistant bacterial pathogens threaten the effectiveness of antimicrobial therapy and complicate the management of infectious diseases worldwide [3,4,5,6,7,8,9,10,11,12,13]. However, the available evidence remains heterogeneous across animal species, production systems, and geographic regions. In many veterinary settings, differences in surveillance methodologies and antimicrobial usage policies limit direct comparisons between studies.

The interconnection between human, animal, and environmental health highlights the necessity of a One Health approach. Evidence indicates that the spread of resistance determinants is accelerated by various factors like anthropogenic activities, including indiscriminate antimicrobial use, inadequate infection control or environmental contamination [10,14,15]. The relationship between antimicrobial use, inappropriate prescribing practices, and the dissemination of resistant bacterial strains has been well documented. In veterinary medicine, this issue is also relevant, as available evidence suggests that antimicrobial prescriptions may not always be fully justified or optimally selected, with relatively high proportions of inappropriate use reported in certain settings (approximately 30–50%) [8,15]. In companion animals, frequent close contact with humans facilitates bidirectional transfer of resistant microorganisms, highlighting the zoonotic potential of veterinary pathogens. Epidemiological studies have identified identical resistance genes and clones in pets and humans, suggesting that companion animals may represent potential reservoirs of clinically relevant resistant bacteria, although the epidemiological importance of direct animal-to-human transmission remains incompletely understood [13,16,17,18,19,20,21,22,23].

In veterinary medicine, antimicrobial agents play a principal role in maintaining animal health and welfare by enabling the treatment and control of bacterial infections. However, inappropriate dosing, prolonged prophylactic use, and the absence of routine antimicrobial susceptibility testing (AST) exacerbate resistance development [24]. Surveillance studies demonstrate that livestock and companion animals are significant reservoirs for MDR bacteria, including methicillin-resistant *Staphylococcus* spp., ESBL-producing *Enterobacteriaceae*, and multidrug-resistant *Pseudomonas aeruginosa* [22,25].

The One Health concept emphasizes the interconnectedness of human, animal, and environmental health and provides a comprehensive framework for addressing antimicrobial resistance [10,19,26,27]. Integrated interventions across different spheres of life sectors are critical for mitigating the emergence and spread of resistant bacteria. Strategies include genomic surveillance, targeted stewardship programs, biosecurity enhancement, and education of healthcare professionals and animal caregivers [28].

The aim of this review is to bring together and critically reassess recent data on AMR in veterinary bacterial pathogens, with a focus on how resistance mechanisms, epidemiological trends, and zoonotic aspects are linked within a One Health framework. Although AMR has been extensively studied, much of the available literature remains fragmented, often addressing these components separately.

Rather than treating these dimensions in isolation, the present work seeks to go beyond a descriptive summary by connecting molecular findings with real-world patterns of spread and transmission. It also examines the role of mobile genetic elements, the limitations of current surveillance systems, and the uncertainties surrounding transmission between animals and humans.

By integrating these perspectives, the review aims to provide a clearer and more practical understanding of how AMR develops and spreads in veterinary settings. In addition, it highlights gaps in current knowledge and outlines directions for more consistent surveillance and more effective control strategies adapted to the One Health context.

This review was developed with the intention of moving beyond a purely descriptive overview of antimicrobial resistance in veterinary bacterial pathogens. The available evidence is not equally strong across all aspects of this topic, and we have therefore tried to separate well-established conclusions from those that remain more uncertain. While the link between antimicrobial exposure and resistance development is well supported, the importance of specific animal reservoirs, environmental sources, and direct animal-to-human transmission is often harder to define. This is partly because many studies differ in design, scale, and resolution, making direct comparisons difficult. These limitations are considered throughout the review, especially when discussing regional variability and the need for longitudinal genomic data to better understand how resistance spreads across animal, human, and environmental interfaces.

## 2. Materials and Methods

The present review was conducted using a structured literature search to identify relevant publications on AMR in veterinary bacterial pathogens, with particular emphasis on resistance patterns, zoonotic transmission, and One Health implications.

The literature search was performed in PubMed, Scopus, and Web of Science. Publications from 2000 to 2025 were considered, with priority given to studies published after 2015, in order to include the most recent evidence. The search terms included different combinations of the following keywords: “antimicrobial resistance”, “veterinary pathogens”, “zoonotic transmission”, “One Health”, “antimicrobial use”, “multidrug resistance”, “resistance genes”, and “horizontal gene transfer”.

Studies were selected according to their relevance to veterinary bacterial antimicrobial resistance, scientific quality, and contribution to the understanding of resistance mechanisms, epidemiological trends, zoonotic transmission pathways, clinical implications, and control strategies. Priority was given to peer-reviewed original articles, systematic reviews, meta-analyses, and reports from international organizations, including the World Health Organization (WHO), World Organisation for Animal Health (WOAH), and European Food Safety Authority (EFSA).

Studies were excluded if they were not directly related to veterinary bacterial AMR, if they focused exclusively on human medicine without a relevant One Health or veterinary component, or if they lacked information on resistance mechanisms, epidemiology, multidrug resistance, zoonotic relevance, environmental dissemination, or clinical applicability.

As this manuscript was designed as a narrative review, the literature was synthesized qualitatively to connect molecular, epidemiological, clinical, and One Health perspectives on antimicrobial resistance in veterinary bacterial pathogens.

## 3. Antimicrobial Use and Emergence of Resistance in Veterinary Medicine

Antimicrobial agents are widely used in veterinary medicine for therapeutic, prophylactic, and, in some cases, metaphylactic purposes. In many countries, veterinary antimicrobial use surpasses human use per kilogram of body mass, largely driven by prophylactic administration in livestock production systems. High-density animal farming further facilitates the rapid dissemination of resistance determinants among bacterial populations [29,30].

The development of antimicrobial resistance is primarily a consequence of selective pressure exerted by antimicrobial exposure [31,32]. Sublethal doses of antimicrobials, commonly resulting from underdosing or incomplete treatment, favor the survival of resistant subpopulations and promote adaptive responses such as biofilm formation and efflux pump overexpression. Recent metagenomic studies reveal that even low-level environmental contamination with veterinary antimicrobials can maintain reservoirs of resistant bacteria in soil and water, creating persistent ecological niches for AMR propagation [33].

Additional contributing factors include the overuse of broad-spectrum antibiotics, empirical therapy without AST guidance, poor compliance with dosing regimens, bacterial adaptive mutations, horizontal gene transfer, and contamination of the environment through animal waste, water runoff, and manure application on agricultural land [34,35,36,37].

**Figure 1:**
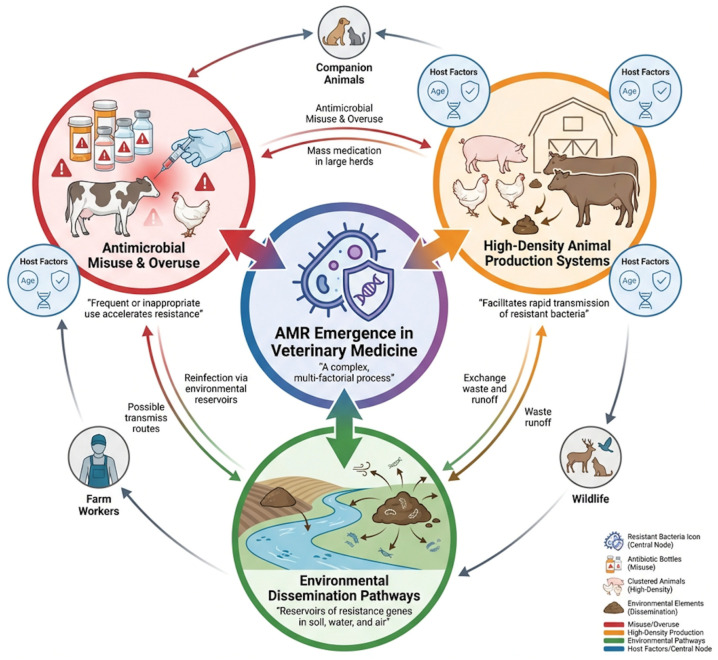
Major factors contributing to the emergence and dissemination of antimicrobial resistance in veterinary medicine.

Figure 1 highlights the multifactorial nature of AMR emergence in veterinary medicine, emphasizing the interaction between antimicrobial exposure, host-related factors, and environmental dissemination pathways.

The commonly used antimicrobial classes used in veterinary medicine, together with their associated resistance trends, are summarized in Table 1.

These data illustrate the widespread occurrence of resistance across multiple antimicrobial classes, emphasizing the need for rational antimicrobial use and routine susceptibility testing in veterinary practice.

The repeated use of these agents, especially in the absence of microbiological guidance, promotes the selection and proliferation of MDR strains. Advanced stewardship programs, incorporating AST and molecular surveillance, are therefore essential to guide rational antimicrobial therapy.

Although the association between antimicrobial exposure and resistance selection is well established, the magnitude of this effect varies substantially between animal species, production systems, antimicrobial classes, and regional prescribing practices. Therefore, resistance trends should be interpreted in relation to local antimicrobial use patterns rather than as uniform global phenomena.

## 4. Antimicrobial Resistance in Major Veterinary Pathogens

Several bacterial species are commonly associated with infections in animals and have demonstrated increasing levels of antimicrobial resistance.

**Figure 2:**
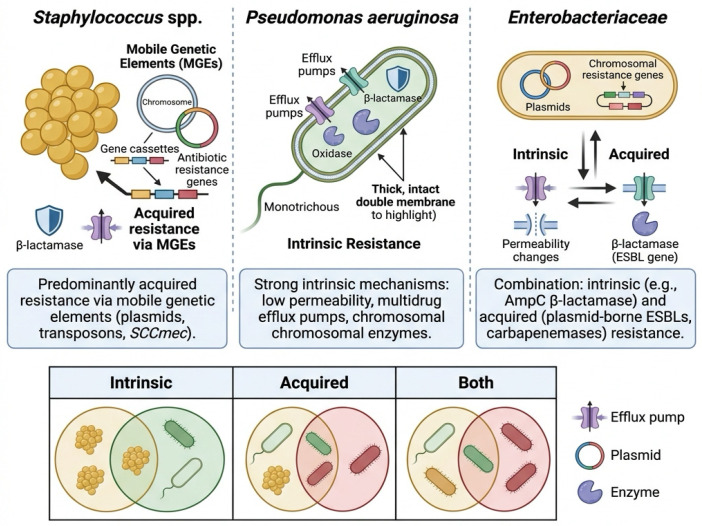
Comparative overview of major bacterial pathogens in veterinary medicine, highlighting their associated infections, key resistance mechanisms, and clinical implications. These pathogens represent significant contributors to antimicrobial resistance in both companion and food-producing animals.

Figure 2 shows comparative overview of major bacterial pathogens in veterinary medicine, highlighting their resistance mechanisms and clinical relevance. *Staphylococcus* spp. predominantly exhibit acquired resistance mediated by mobile genetic elements, *Pseudomonas aeruginosa* displays strong intrinsic resistance mechanisms, while members of the order Enterobacterales combine intrinsic and acquired resistance. Arrows indicate interactions between resistance mechanisms. The lower schematic illustrates the coexistence of intrinsic and acquired resistance within bacterial populations.

The arrows shown in the figure should be interpreted as representing interactions between resistance mechanisms rather than strict linear pathways. In the case of Enterobacterales, the schematic highlights the coexistence of intrinsic resistance mechanisms (e.g., reduced permeability and chromosomal enzymes) and acquired resistance determinants such as plasmid-mediated ESBLs and carbapenemases.

The lower portion of the figure, represented as overlapping circles, illustrates the convergence of intrinsic and acquired resistance mechanisms within bacterial populations. The icons within these areas symbolize different resistance profiles and bacterial populations, rather than specific species. This conceptual representation emphasizes that multidrug resistance frequently arises from the interaction of multiple mechanisms.

### 4.1. Staphylococcus *spp.*

Methicillin-resistant *Staphylococcus aureus* (MRSA) represents a prototypical “superbug” and is associated with a significant worldwide mortality burden due to infections that exhibit resistance to conventional antibiotic therapies [53].

Bacteria belonging to the genus *Staphylococcus*, including *S. aureus* and *S. pseudintermedius*, are frequently isolated from skin infections, otitis, wound infections, and postoperative complications in companion animals [23,54,55,56]. Methicillin-resistant staphylococci (MRSA, MRSP) are highly prevalent in veterinary practice and frequently display multidrug resistance, often associated with the co-selection of resistance determinants linked to several commonly used antimicrobial classes [23,55].

Recent genomic studies demonstrate that mobile genetic elements, such as SCCmec cassettes and plasmids, facilitate the horizontal transfer of resistance determinants between animal and human strains. In addition, biofilm formation and small colony variants contribute to persistent infections and reduced antimicrobial susceptibility, complicating treatment outcomes [57].

### 4.2. Pseudomonas aeruginosa

*Pseudomonas aeruginosa* is an opportunistic Gram-negative bacterium ubiquitous in various places (soil, water, hospital environments) [15,58]. It is commonly associated with otitis externa, urinary tract and wound infections, as well as postoperative complications in companion animals. Its intrinsic resistance to many antimicrobials is mainly related to reduced outer membrane permeability and active efflux systems, with biofilm formation further enhancing persistence. In addition, acquired mechanisms such as β-lactamase production and target site mutations further restrict therapeutic options.

Emerging MDR strains often carry plasmid-mediated resistance genes and integrons, highlighting the potential for rapid dissemination within veterinary settings. These strains may serve as reservoirs for resistance determinants with implications for human health.

### 4.3. Enterobacterales

Members of the order Enterobacterales, including *Escherichia coli*, *Proteus mirabilis*, and *Enterobacter* spp., are important opportunistic pathogens in veterinary medicine. *E. coli* is frequently associated with urinary tract infections, septicemia, and gastrointestinal disease. In contrast, *Proteus mirabilis* and *Enterobacter* spp. are more often involved in urinary tract and wound infections [59].

Also, the increasing detection of ESBL-producing and carbapenemase-producing strains in animals is a major concern. These resistance mechanisms may limit the effectiveness of critically important antimicrobials used in both human and veterinary medicine. Moreover, plasmids and transposons can promote horizontal gene transfer. As a result, resistance traits may spread between bacterial populations and across different host species.

Common antimicrobial resistance patterns observed in major veterinary pathogens are summarized in Table 2.

These patterns highlight the increasing prevalence of multidrug resistance among clinically relevant veterinary pathogens and emphasize the growing challenges in antimicrobial therapy.

However, the relative contribution of each pathogen to antimicrobial resistance burden varies significantly depending on the clinical context and host species. While *Staphylococcus* spp. is predominantly associated with companion animal infections, Gram-negative bacteria such as *Pseudomonas aeruginosa* and *Enterobacteriaceae* pose a greater therapeutic challenge due to their intrinsic resistance mechanisms and higher capacity for acquiring additional resistance determinants. This heterogeneity highlights the need for pathogen-specific surveillance and targeted therapeutic strategies rather than uniform antimicrobial approaches.

Taken together, these findings indicate that the clinical relevance of AMR differs considerably between pathogens. In staphylococci, acquired resistance and clonal dissemination are central concerns, whereas in *P. aeruginosa* and Enterobacterales, intrinsic resistance combined with acquired determinants creates a greater therapeutic challenge. This distinction is important because it argues against a uniform approach to surveillance and treatment.

## 5. Multidrug Resistance in Veterinary Bacterial Isolates

MDR is commonly defined as resistance to at least one antimicrobial agent in three or more antimicrobial classes [66]. The emergence of MDR bacteria represents a significant challenge for both veterinary and human medicine [67].

The prevalence of multidrug resistance among pathogens affecting both humans and animals is increasing progressively on a global scale. Moreover, these patterns of resistance are expanding to include commensal microbiota derived from human, animal, and environmental sources, thereby constituting a significant threat to public health [67,68].

Recent studies have shown that MDR often arises from the accumulation of multiple resistance genes within mobile genetic elements such as plasmids, transposons, and integrons. These elements facilitate horizontal gene transfer between bacterial populations, increasing the speed at which resistance spreads in both companion and food-producing animals. MDR pathogens in livestock can impact productivity and economic outcomes, while in companion animals they complicate clinical management and increase the risk of zoonotic transmission. Environmental reservoirs, including wastewater and soil contaminated with animal excreta, also contribute to the maintenance and dissemination of MDR bacteria [69,70].

Continuous monitoring of antimicrobial susceptibility patterns is therefore essential for identifying emerging MDR strains and supporting the development of effective treatment strategies.

Despite increasing awareness, current surveillance systems remain fragmented and often lack standardization across regions and animal species. This limitation hampers accurate comparisons between studies and may underestimate the true global burden of multidrug resistance in veterinary settings. Consequently, harmonized methodologies and longitudinal studies are essential to better understand MDR dynamics and inform effective intervention strategies.

## 6. Epidemiology and Zoonotic Transmission of AMR

### 6.1. Epidemiological Trends in Companion Animals and Livestock

Global prevalence of MDR pathogens in companion animals and livestock has risen sharply over the last decade [71,72].

Regional differences reflect variations in antimicrobial usage policies, veterinary practices, and biosecurity measures [73]. Longitudinal surveillance demonstrates that countries with strict antimicrobial regulations and active stewardship programs exhibit lower prevalence of MDR strains. Molecular epidemiology further confirms interspecies and environmental dissemination of identical resistance genes, highlighting the necessity for coordinated One Health surveillance [74,75,76].

Emerging trends indicate an increase in carbapenem-resistant *Enterobacteriaceae* and colistin-resistant *E. coli* in livestock, emphasizing the importance of global monitoring, risk assessment, and proactive intervention.

However, reported prevalence rates should be interpreted with caution, as differences in sampling strategies, diagnostic methodologies, and regional antimicrobial usage policies can significantly influence results. These inconsistencies highlight the need for standardized surveillance frameworks to enable reliable global comparisons.

### 6.2. Zoonotic Transmission of Antimicrobial-Resistant Bacteria

#### 6.2.1. Transmission Pathways Between Animals, Humans, and the Environment

The zoonotic transmission of antimicrobial-resistant bacteria represents an important concern for public health. Animals carrying resistant microorganisms may serve as reservoirs capable of transmitting these bacteria to humans through direct or indirect contact.

It is believed that around 60% of pathogens impacting humans, along with nearly 75% of newly emerging human diseases, have a zoonotic origin. Throughout history, many severe human infections have arisen from animal sources [77,78].

Zoonotic transmission of antimicrobial-resistant bacteria should be interpreted with caution. It is important to distinguish between: (i) direct transmission events demonstrated through epidemiological or genomic evidence; (ii) the presence of shared resistance genes or bacterial clones in animal and human populations; (iii) reverse transmission (human-to-animal); and (iv) indirect transmission through environmental reservoirs. While similarities between animal and human isolates are frequently reported, direct transmission is less commonly demonstrated and often requires high-resolution genomic confirmation.

**Figure 3:**
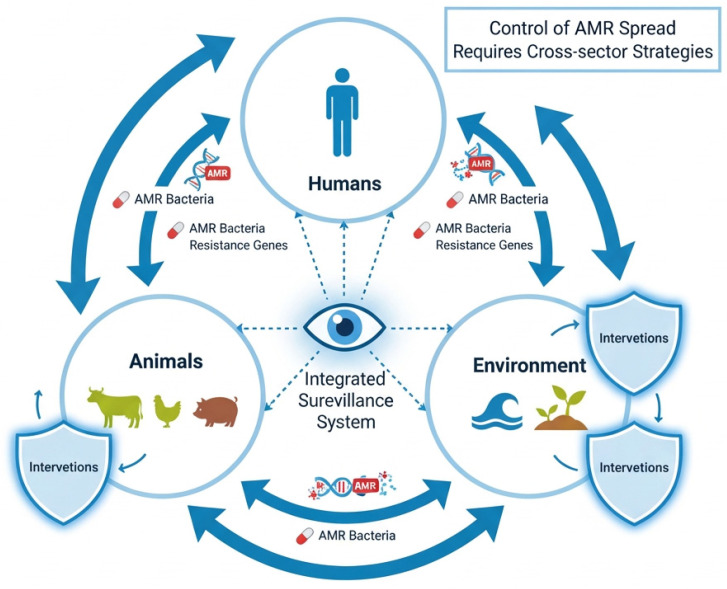
One Health transmission pathways of antimicrobial-resistant bacteria. Resistant microorganisms and genes circulate between humans, animals, and the environment through direct contact, food chains, and environmental contamination, highlighting the interconnected nature of AMR dissemination.

Figure 3 illustrates the interconnected circulation of resistant bacteria and resistance genes between animals, humans, and environmental reservoirs within the One Health framework.

Epidemiological surveillance has detected identical resistance genes and bacterial clones in humans and animals, indicating bidirectional transfer. Companion animals in close contact with humans are particularly important in this context. Farm workers handling livestock, and veterinarians in clinical settings, are at increased risk of colonization or infection. Environmental contamination of veterinary clinics, animal bedding, and wastewater can further facilitate the spread of resistant bacteria. These findings underscore the need for integrated One Health surveillance programs that monitor both human and animal populations to mitigate the risk of zoonotic AMR transmission [77].

Although numerous studies report the presence of identical resistance genes in humans and animals, the directionality of transmission remains incompletely understood. While some evidence supports zoonotic transfer from animals to humans, other studies suggest reverse transmission or shared environmental sources as primary drivers []. This heterogeneity highlights the limitations of current epidemiological approaches and underscores the need for longitudinal, high-resolution genomic studies to clarify transmission pathways [12,26,28,70].

Furthermore, most available data are derived from cross-sectional studies, which limit the ability to establish causal relationships or determine the directionality of transmission. High-resolution genomic approaches, including whole-genome sequencing, are increasingly required to accurately track transmission pathways and differentiate between zoonotic, reverse zoonotic, and environmentally driven dissemination.

#### 6.2.2. Companion Animals and Livestock as Reservoirs of Resistant Bacteria

Pets, livestock, and farmed animals can contribute to the maintenance and spread of MDR bacteria, but their role as reservoirs should be interpreted in relation to the bacterial species involved, the intensity of human–animal contact, and the quality of the supporting epidemiological evidence. Human exposure can occur via direct contact, contaminated surfaces, or animal-derived food products. Molecular epidemiology confirms shared clones and resistance genes between humans and animals. Effective interventions include hygiene measures, AST-guided therapy, and surveillance programs across human and animal populations.

In recent years, dogs and cats have been progressively recognized as potential reservoirs and significant routes of transmission. The transfer of antimicrobial resistance from dogs and cats to humans has been documented in the scientific literature; however, the overall magnitude of this process remains largely undetermined. In Europe, it is estimated that over 102 million households own cats, while approximately 86 million households keep dogs [78].

Dogs and cats often carry MRSP, MRSA, and ESBL-producing Enterobacteriaceae without showing symptoms [79,80,81]. Colonized pets may facilitate human exposure to resistant bacteria, particularly in households and veterinary settings with close and repeated contact, although direct transmission requires confirmation through epidemiological and genomic evidence. Preventive measures include routine screening in clinical settings, proper hygiene, and judicious antimicrobial use. Longitudinal studies show that pets can intermittently shed MDR bacteria over months, highlighting the importance of surveillance [17].

## 7. One Health and Environmental Dimensions of AMR

### 7.1. One Health Framework for AMR Control

The One Health concept emphasizes the interconnectedness of human, animal, and environmental health and provides a comprehensive framework for addressing antimicrobial resistance. The spread of resistant bacteria across different ecological compartments demonstrates that antimicrobial resistance cannot be effectively controlled through isolated interventions.

Zoonosis-related and newly arising infectious conditions necessitate timely and coordinated responses, which can be effectively achieved only through the implementation of One Health–oriented frameworks that systematically address anticipated challenges and integrate evidence-based, context-specific solutions [82].

Successful One Health strategies integrate veterinary, human, and environmental surveillance with targeted stewardship programs, infection prevention measures, and public education. Coordination between medical and veterinary professionals is crucial to identify high-risk pathogens, implement biosecurity measures, and reduce the misuse of antimicrobials. Environmental management, including proper disposal of animal waste and treatment of farm effluents, minimizes the dissemination of resistance genes into the wider ecosystem. Rapid molecular diagnostics and genomic surveillance provide timely data to track MDR clones, enabling evidence-based interventions [82,83,84,85].

**Figure 4:**
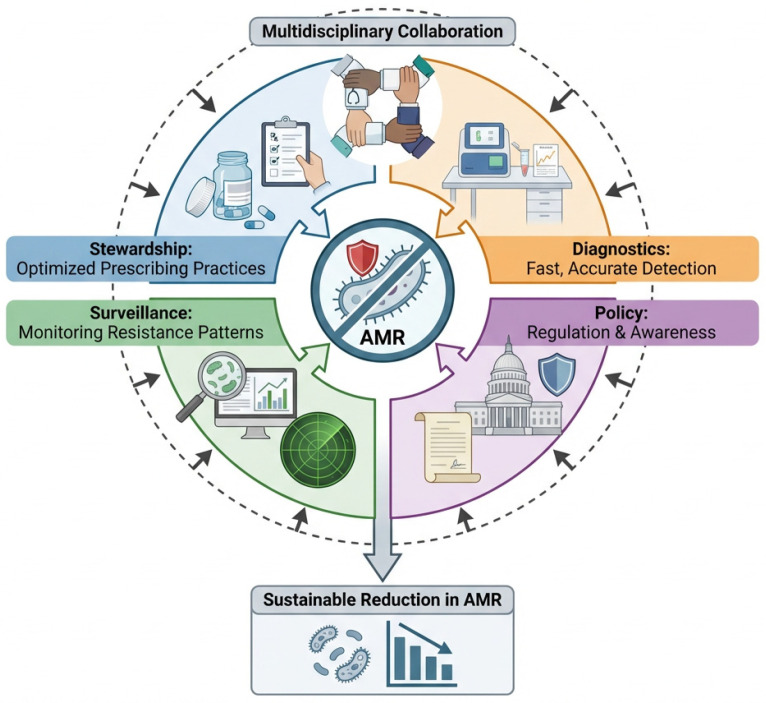
Integrated One Health strategies for controlling antimicrobial resistance, including stewardship programs, surveillance systems, diagnostics, and policy interventions.

Figure 4 highlights the key components of effective AMR control, including antimicrobial stewardship, surveillance systems, rapid diagnostics, clinical decision-making, and policy interventions. Within this framework, rapid and accurate diagnosis represents a central hub, linking antimicrobial management with surveillance and treatment decisions. The integration of these strategies emphasizes the need for multidisciplinary collaboration to achieve sustainable reductions in antimicrobial resistance.

### 7.2. Environmental Reservoirs and Dissemination Pathways

The environment plays a central role in the dissemination and maintenance of AMR. Manure, farm runoff, and untreated effluents can introduce MDR bacteria and mobile genetic elements into soil and water. Antimicrobials and heavy metals present in agricultural waste exert selective pressure on environmental microbiota, promoting the persistence and evolution of resistance [86,87].

Environmental monitoring has detected ESBL-producing *E. coli*, MRSA, and carbapenem-resistant *Enterobacteriaceae* in surface water and agricultural soil, demonstrating the potential for horizontal gene transfer to wildlife and human commensals. Mitigation strategies include proper manure management, wastewater treatment, controlled use of antimicrobials, and surveillance of AMR in environmental compartments. Integration of environmental interventions into One Health programs is critical for reducing ecological reservoirs of MDR bacteria [88,89].

## 8. Molecular Mechanisms of Antimicrobial Resistance in Veterinary Pathogens

Bacteria employ multiple molecular strategies to evade antimicrobial therapy. In *Staphylococcus* spp., the *mec*A gene encodes penicillin-binding protein *PBP2a*, which lowers affinity for β-lactams, leading to methicillin resistance. Additional resistance determinants include genes conferring resistance to macrolides (*erm*A, *erm*C), tetracyclines (*tet*K, *tet*M), and aminoglycosides (*aac*(6′)-*Ie-aph*(2″)-*Ia*).

**Figure 5:**
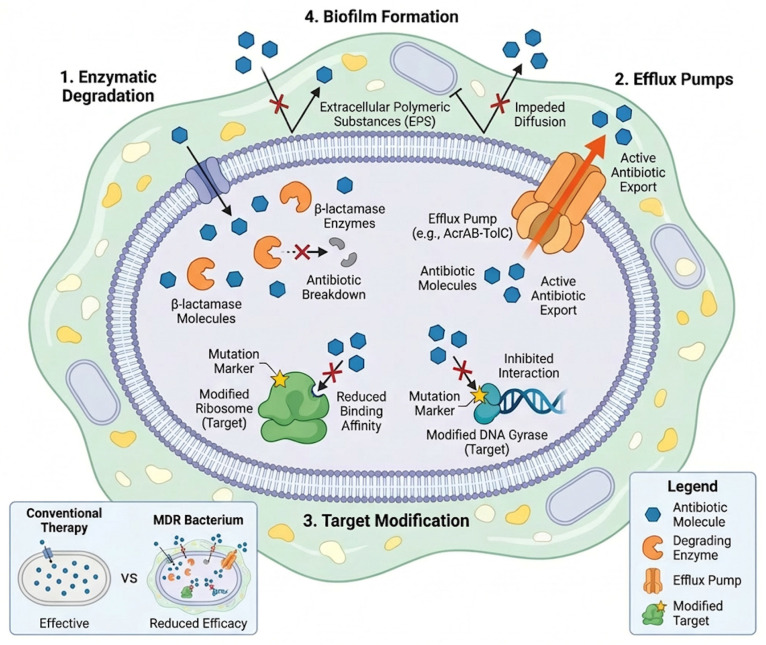
Molecular mechanisms of antimicrobial resistance in veterinary bacterial pathogens. Key mechanisms include enzymatic degradation of antibiotics, efflux pumps, target modification, horizontal gene transfer, and biofilm formation, all contributing to reduced antimicrobial efficacy.

The principal molecular mechanisms underlying antimicrobial resistance in veterinary pathogens are illustrated in Figure 5.

Figure 5 demonstrates that multiple resistance mechanisms, including enzymatic degradation, efflux pumps, target modification, and biofilm formation, often coexist within the same bacterial strain. This complexity explains the rapid emergence of multidrug-resistant phenotypes and the reduced effectiveness of conventional antimicrobial therapies.

Resistance to antimicrobial agents in staphylococci is predominantly mediated by the *mec*A gene, which confers reduced susceptibility to the broad spectrum of β-lactam antibiotics. This gene is embedded within a mobile genetic element designated as the staphylococcal cassette chromosome mec (SCCmec). The SCCmec element integrates at a highly conserved chromosomal insertion site located at the 3′ terminus of the orfX locus, a gene responsible for encoding an RNA methyltransferase. The acquisition of *mec*A significantly contributes to the adaptive success of staphylococcal populations, providing a selective advantage in diverse ecological niches, including healthcare facilities, veterinary environments and community settings [90].

The *mec*A and *mec*C genetic determinants are responsible for the synthesis of the penicillin-binding proteins *PBP2*A and *PBP2*A′, respectively. These proteins exhibit markedly reduced binding affinity toward the majority of β-lactam antibiotics, thereby diminishing the effectiveness of this antibiotic class [91].

Penicillin-binding proteins (PBPs) play a crucial role in the terminal steps involving bacterial cell wall peptidoglycan (PG) synthesis and serve as the principal molecular targets for penicillin as well as other β-lactam antibiotics. In most cases, methicillin resistance arises through the acquisition of an alternative penicillin-binding protein, PBP2A, which can substitute for the functions of native PBPs because it displays a markedly reduced affinity for β-lactam antibiotics [92]. The production of these exogenous PBPs enables bacterial cells to maintain cell wall synthesis even when exposed to elevated concentrations of the antibiotic [91].

In *P. aeruginosa*, antimicrobial resistance results from the interaction of intrinsic, acquired, and adaptive mechanisms. Intrinsic resistance is mainly related to low outer membrane permeability, chromosomal AmpC β-lactamase activity, multidrug efflux pumps, and biofilm formation. Acquired resistance may involve additional β-lactamases, aminoglycoside-modifying enzymes, mutations affecting fluoroquinolone targets, and mobile genetic elements such as integrons carrying resistance gene cassettes. The coexistence of these mechanisms contributes to multidrug-resistant phenotypes and limits therapeutic options in veterinary infections [93,94].

In *Enterobacteriaceae*, resistance is frequently mediated by extended-spectrum β-lactamases (ESBLs), *Amp*C β-lactamases, and carbapenemases, often carried on conjugative plasmids. Horizontal gene transfer facilitated by plasmids, integrons, and transposons accelerates dissemination of multidrug resistance across species and ecological niches. Biofilm formation and the presence of persister cells further enhance survival under antimicrobial pressure [63,65,67].

Recent genomic studies show co-localization of virulence and resistance genes on mobile genetic elements, creating highly adapted MDR clones capable of colonizing animals and humans. Understanding these molecular mechanisms is crucial for designing targeted interventions and novel therapeutics.

Although these mechanisms are well characterized at the molecular level, their relative contribution to clinical resistance phenotypes may vary depending on environmental conditions and host factors. In many cases, the interaction between multiple resistance mechanisms rather than a single determinant is responsible for therapeutic failure, complicating both diagnosis and treatment strategies.

## 9. Antimicrobial Stewardship and One Health-Based Control Strategies

Antimicrobial stewardship programs (ASP) optimize therapeutic outcomes, reduce unnecessary antimicrobial use, and curb the emergence of MDR bacteria. Key components include AST-guided therapy, restriction of critically important antibiotics, prescriber feedback, and owner education. Digital monitoring systems, such as prescription tracking and decision support tools, enhance compliance and provide real-time data on antimicrobial usage trends [95,96].

In food-producing animals, stewardship strategies involve vaccination, biosecurity enhancements, herd health management, and careful selection of therapeutic agents. Evidence demonstrates that ASP implementation reduces overall antimicrobial consumption and decreases resistance prevalence, improving animal welfare and public health outcomes. Collaborative training programs for veterinarians, farmers, and pet owners are essential to ensure sustainability of stewardship efforts.

Although antimicrobial stewardship programs have demonstrated effectiveness in reducing antimicrobial use, their implementation in veterinary settings is often inconsistent. Variability in regulatory frameworks, limited access to diagnostic tools, and economic pressures in livestock production systems hinder widespread adoption. This disparity may contribute to persistent regional differences in AMR prevalence.

In addition, the effectiveness of antimicrobial stewardship programs is highly dependent on compliance at both the practitioner and owner level. Behavioral and economic factors often influence prescribing practices, suggesting that successful implementation requires not only regulatory measures but also educational and socio-economic interventions.

Human behavior plays a critical role in the emergence and spread of AMR. Owners may administer incomplete courses of antibiotics, self-medicate pets, or demand antimicrobials for non-bacterial conditions. In livestock production, prophylactic or growth-promoting antimicrobial use persists in some regions due to economic incentives [79].

Educational interventions targeting veterinarians, farmers, and pet owners improve awareness of AMR risks and promote adherence to AST-guided therapy. Public campaigns, training workshops, and behavioral nudges can reduce inappropriate antimicrobial use, enhance biosecurity, and support One Health strategies to mitigate resistance emergence [97,98].

In resource-limited settings, surveillance strategies should prioritize high-impact and cost-effective approaches. Sentinel surveillance targeting key bacterial pathogens such as *Escherichia coli*, *Salmonella* spp., and *Staphylococcus aureus* in food-producing animals may provide valuable early warning signals with relatively low resource requirements. Additionally, simplified antimicrobial use (AMU) recording systems at the farm level can improve monitoring and guide targeted interventions.

## 10. Clinical Management and Alternative Strategies for MDR Infections

### 10.1. Clinical Challenges in Treating MDR Infections

MDR infections significantly complicate veterinary clinical practice. Treatment failures, prolonged therapy, and increased costs are common consequences. Biofilms, intracellular survival, and persisted cell populations reduce antimicrobial efficacy, while limited therapeutic options in companion and food animals increase reliance on critically important antibiotics [99,100].

Novel interventions under investigation include bacteriophage therapy, antimicrobial peptides, immunomodulatory agents, and combination therapies. Integration of these strategies with conventional antimicrobial regimens may reduce selection pressure, prevent resistance development, and improve clinical outcomes in MDR infections [101,102].

While alternative strategies such as bacteriophage therapy, antimicrobial peptides, and CRISPR-based approaches show considerable promise, their translation into routine veterinary practice remains limited. Challenges related to regulatory approval, host specificity, delivery mechanisms, and cost-effectiveness continue to restrict large-scale implementation. Consequently, these approaches should currently be viewed as complementary rather than substitutive to conventional antimicrobial therapy [103,104].

Moreover, the clinical efficacy of these alternative therapies remains insufficiently validated in large-scale veterinary studies. Most available data are derived from experimental or limited clinical settings, highlighting the need for robust clinical trials before widespread implementation.

### 10.2. Alternative Strategies to Combat AMR

Non-antibiotic approaches are gaining importance as adjunct or alternative therapies. Phage therapy demonstrates species-specific lytic activity against MDR pathogens, including MRSP and ESBL-producing *E. coli* [105,106]. Antimicrobial peptides disrupt bacterial membranes and biofilms, enhancing susceptibility to conventional antibiotics [107]. Probiotics and competitive exclusion strategies modify host microbiota to limit pathogen colonization [108].

Emerging technologies, such as nanotechnology-based drug delivery and CRISPR (Clustered Regularly Interspersed Short Palindromic Repeats)- Cas antimicrobials, enable targeted elimination of resistance genes, reducing off-target effects and minimizing ecological impact. Integration of these approaches with stewardship programs may significantly reduce antimicrobial reliance and slow MDR propagation [108,109,110,111,112].

**Figure 6:**
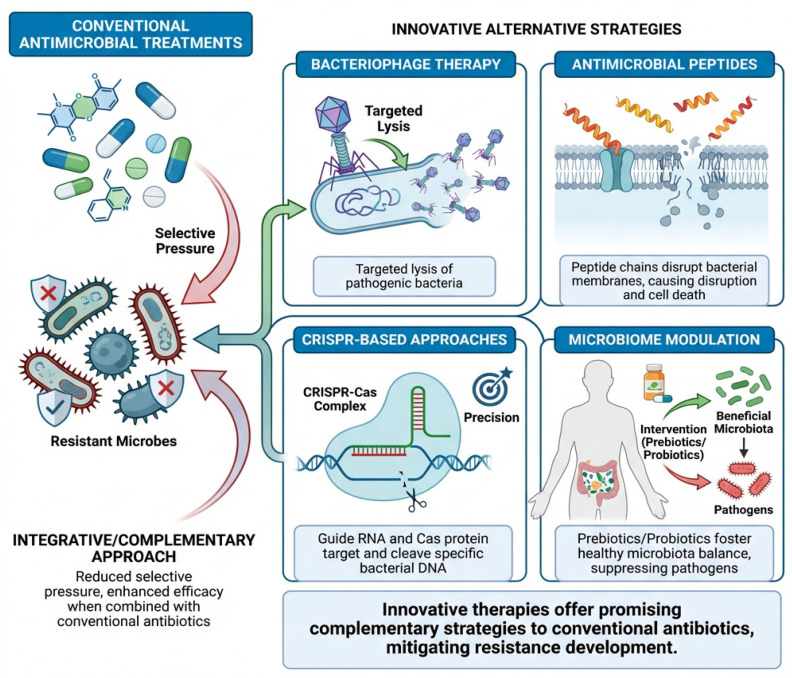
Emerging alternative strategies to combat antimicrobial resistance include phage therapy, antimicrobial peptides, CRISPR-based approaches, and microbiome modulation.

Figure 6 highlights innovative therapeutic strategies, including bacteriophage therapy, antimicrobial peptides, CRISPR-based approaches, and microbiome modulation. These alternatives demonstrate significant potential to complement conventional antimicrobial treatments and reduce selective pressure driving resistance.

Despite their promising potential, these alternative strategies are unlikely to fully replace conventional antimicrobials in the near future. Instead, their greatest value may lie in combination therapies and integrated approaches aimed at reducing antimicrobial use and slowing resistance development.

### 10.3. Diagnostic Innovations in Veterinary AMR

Rapid molecular diagnostics, such as PCR, qPCR, and whole-genome sequencing, allow early identification of resistance genes and MDR strains. Point-of-care tests in clinics and farms guide AST-informed therapy. Bioinformatic analyses track resistance gene dissemination across populations and environments, informing intervention strategies [113,114,115].

## 11. Global Policy Initiatives and Future Directions in Veterinary AMR

International organizations, including WHO, OIE, and FAO, support One Health approaches, harmonized surveillance, and regulatory policies to combat AMR. Restricting critically important antimicrobials, mandating AST-based prescriptions, and promoting stewardship programs have proven effective in reducing AMR prevalence in several countries. Collaborative initiatives improve data sharing, risk assessment, and coordinated interventions [116].

Future research should focus on: (i) molecular epidemiology and genomic surveillance of MDR strains, (ii) environmental pathways of AMR dissemination, (iii) rapid diagnostics and alternative therapeutics, (iv) optimization of stewardship programs, and (v) integration of One Health principles into veterinary policy and practice. Understanding the interplay between host, pathogen, antimicrobial use, and environment is critical to predict and prevent emerging resistance threats [117].

Beyond regulatory frameworks and education, improving compliance among farmers and veterinarians requires incentive-based approaches. These may include certification schemes for farms adhering to antimicrobial stewardship principles, financial subsidies or tax reductions for responsible antimicrobial use, and market-based incentives such as premium pricing for products derived from low-AMU systems. Such strategies can enhance adherence to guidelines and facilitate the implementation of sustainable AMR control programs.

## 12. Economic Implications of Antimicrobial Resistance in Veterinary Medicine

Beyond its clinical and public health relevance, AMR also has important economic consequences for veterinary medicine, animal production, food security, and One Health-based disease control. Currently, infections caused by antimicrobial-resistant pathogens are responsible for approximately 700,000 fatalities annually across the globe. If left uncontrolled, projections indicate that these pathogens could result in 10 million lives lost each year, with an associated cumulative economic burden estimated at $100 trillion by 2050 [118,119,120].

Analogous to AMU in human medicine, the application of antimicrobials in animal agriculture may facilitate the emergence of AMR among both human and animal pathogenic populations, thereby generating substantial health and economic burdens for society. Furthermore, such burdens are characterized in economic theory as externalities, indicating that they arise as unintended consequences of AMU, are borne by society at large, and are insufficiently incorporated into antimicrobial pricing, thus justifying the need for policy intervention [121].

In the long run, the unregulated application of antimicrobial agents and the resulting escalation of antimicrobial resistance may generate substantially higher economic burdens worldwide. As resistance proliferates, increasing antimicrobial usage ceases to ensure effective disease management, thereby compromising both livestock productivity and the reliability of food supply systems [122].

MDR infections impose substantial economic burdens on veterinary healthcare and animal production. Increased treatment costs, prolonged hospitalization, and reduced productivity in livestock contribute to financial losses. In companion animals, MDR infections require costly alternative therapies, extended care, and increased veterinary interventions [121].

Globally, economic modeling estimates that AMR in livestock and companion animals results in billions of dollars in direct and indirect costs annually. Preventive measures, stewardship programs, and alternative therapeutics not only improve clinical outcomes but also offer significant cost savings by reducing disease burden and limiting antimicrobial consumption [121].

From this perspective, the economic burden of AMR strengthens the case for prevention-oriented veterinary practice. Reducing unnecessary antimicrobial use is not only a clinical priority, but also a way to limit the costs associated with MDR infections. This is especially relevant in food-producing animals, where resistance can affect productivity as well as animal health. Economic evidence may therefore help support One Health policies focused on earlier detection, better management, and more responsible antimicrobial use.

## 13. Conclusions and Recommendations

Antimicrobial resistance in veterinary bacterial pathogens is a multifactorial challenge with important implications for animal health, public health, food production, and environmental sustainability. The increasing occurrence of multidrug-resistant bacteria, particularly among *Staphylococcus* spp., *Pseudomonas aeruginosa*, and *Enterobacteriaceae*, continues to complicate clinical management and limit therapeutic options.

The widespread use and misuse of antimicrobials in veterinary medicine continue to drive the selection and dissemination of resistant strains. In this context, integrated strategies based on antimicrobial stewardship, routine susceptibility testing, and improved biosecurity are essential to mitigate resistance development.

The One Health framework plays a central role in addressing AMR by recognizing the interconnectedness of human, animal, and environmental health. Effective control requires coordinated efforts between veterinarians, researchers, policymakers, and public health authorities to monitor resistance trends, reduce antimicrobial misuse, and limit zoonotic transmission.

In addition, emerging alternative approaches, including bacteriophage therapy, antimicrobial peptides, and microbiome-based strategies, offer promising perspectives for reducing reliance on conventional antimicrobials. Continued research and innovation in these areas are essential to support sustainable AMR management.

Overall, veterinary AMR should be viewed as a problem that extends beyond the treatment of individual infections. Resistant bacteria can move between animals, people, and the environment, while the way antimicrobials are used in practice influences both clinical outcomes and wider public health risks. For this reason, control measures need to be coordinated rather than isolated. A stronger response will depend on better surveillance, more responsible antimicrobial use, closer attention to environmental sources of resistance, and One Health policies that are guided by evidence and adapted to real veterinary settings.

## Figures and Tables

**Table 1:** Antimicrobial classes commonly used in veterinary medicine and resistance trends.

Antimicrobial Class	Examples Commonly Used in Veterinary Medicine	Major Bacterial Targets	Frequently Reported Resistance Trends
β-lactams	Amoxicillin, ampicillin, cephalexin, ceftiofur	*Staphylococcus* spp., *E. coli*, *Proteus* spp.	Increasing resistance reported in multiple regions including Europe and Asia, particularly in livestock-associated isolates [38,39]
Fluoroquinolones	Enrofloxacin, marbofloxacin, ciprofloxacin	*Pseudomonas aeruginosa*, *E. coli*, *Enterobacter* spp.	Rising resistance in Gram-negative bacteria across companion and food animals in regions with high antimicrobial usage [40]
Aminoglycosides	Gentamicin, amikacin, neomycin	*Pseudomonas aeruginosa*, *E. coli*, *Staphylococcus* spp.	Moderate but increasing resistance in hospital and veterinary clinical isolates [41]
Tetracyclines	Tetracycline, doxycycline, oxytetracycline	*E. coli*, *Staphylococcus* spp.	High resistance prevalence globally, especially in intensive livestock production systems [42]
Macrolides	Erythromycin, tylosin, tilmicosin	*Staphylococcus* spp., respiratory pathogens	Increasing resistance in Gram-positive pathogens, particularly in Europe and North America [43]
sulfonamide-diaminopyrimidine combinations	Sulfamethoxazole-trimethoprim	*E. coli*, *Proteus mirabilis*	Widespread resistance reported globally with regional variability depending on antimicrobial usage practices [44]
Phenicols	Florfenicol, chloramphenicol	Respiratory and systemic pathogens	Variable resistance patterns, higher in regions with intensive livestock farming [45,46]
Polymyxins	Colistin	Gram-negative bacteria, especially *E. coli*	Emergence of resistance reported in livestock, raising significant One Health concerns [47]
Lincosamides	Lincomycin, clindamycin	*Staphylococcus* spp., anaerobes	Variable resistance reported, more common in companion animals [48]
Nitroimidazoles	Metronidazole	Anaerobic bacteria	Generally low resistance, but regional variability exists [49]
Pleuromutilins	Tiamulin, valnemulin	Gram-positive respiratory pathogens	Low to moderate resistance, mainly in food-producing animals [50]
Streptogramins	Virginiamycin	Gram-positive bacteria (*Staphylococcus* spp., *Enterococcus* spp.)	Resistance reported, often associated with cross-resistance to macrolides and lincosamides [51].
Cyclic polypeptides	Bacitracin	Gram-positive bacteria	Resistance reported in veterinary isolates [52]

Table 1 provides a context-dependent summary of commonly used antimicrobial classes in veterinary medicine. Resistance patterns are synthesized from published studies and demonstrate substantial geographic and temporal variability influenced by antimicrobial usage practices, animal production systems, and regional stewardship policies. Therefore, the reported trends should be interpreted as general patterns rather than absolute global values.

**Table 2:** Common AMR patterns in major veterinary pathogens.

Bacterial Pathogen	Common Infections in Animals	Frequently Reported Resistance	Clinical Implications
*Staphylococcus* spp.	Skin, otitis, wounds, postoperative infections	Resistance to β-lactams (including methicillin), macrolides, tetracyclines, and fluoroquinolones widely reported in companion animals [60]	MDR complicates treatment in companion animals
*Pseudomonas aeruginosa*	Otitis externa, wound infections, urinary tract infections	Intrinsic resistance to multiple classes; acquired resistance to β-lactams, fluoroquinolones, and aminoglycosides reported in clinical isolates [61]	Intrinsic + acquired resistance limits therapy
*E. coli*	Urinary tract infections, septicemia, gastrointestinal infections	High prevalence of resistance to β-lactams, fluoroquinolones, tetracyclines, and sulfonamides; ESBL-producing strains reported globally [62,63]	Increasing prevalence of MDR isolates in animals
*Proteus mirabilis*	Urinary tract infections, wound infections	Resistance to β-lactams, fluoroquinolones, and aminoglycosides reported, with regional variability [64]	Emerging resistance patterns reported in veterinary isolates
*Enterobacter* spp.	Opportunistic infections, urinary tract infections	Resistance to β-lactams (including AmpC), cephalosporins, and fluoroquinolones reported in veterinary isolates [65]	Intrinsic + acquired resistance contributes to treatment difficulties

## Data Availability

No new data were created or analyzed in this study.

## Abbreviations

The following abbreviations are used in this manuscript:

AMR	Antimicrobial resistance
MDR	Multidrug-resistant
AST	Antimicrobial susceptibility testing
MRSA	Methicillin-resistant *Staphylococcus aureus*
ASP	Antimicrobial stewardship programs
AMU	Antimicrobial use
WHO	World Health Organization
WOAH	World Organisation for Animal Health
EFSA	European Food Safety Authority
